# Ethical interventions: revisiting the assessment tools for health-system pharmacists

**DOI:** 10.3389/fmed.2025.1536044

**Published:** 2025-04-11

**Authors:** Hong Tham Pham, Ronald L. Castelino, Tyree H. Kiser, Toby C. Trujillo, Megan K. Fischer, Kim-Huong Truong-Nguyen, Bao-Chau Truong-Nguyen, Minh-Hoang Tran

**Affiliations:** ^1^Faculty of Pharmacy, Nguyen Tat Thanh University, Ho Chi Minh City, Vietnam; ^2^School of Pharmacy, Faculty of Medicine and Health, The University of Sydney, Darlington, NSW, Australia; ^3^Pharmacy Department, Blacktown Hospital, Blacktown, NSW, Australia; ^4^Department of Clinical Pharmacy, Skaggs School of Pharmacy and Pharmaceutical Sciences, University of Colorado Anschutz Medical Campus, Aurora, CO, United States; ^5^Department of Pharmacy, University of Colorado Hospital, UCHealth, Aurora, CO, United States; ^6^Department of Pharmacy, Nhan Dan Gia Dinh Hospital, Ho Chi Minh City, Vietnam; ^7^Faculty of Pharmacy, Pham Ngoc Thach University of Medicine, Ho Chi Minh City, Vietnam; ^8^NTT Hi-Tech Institute, Nguyen Tat Thanh University, Ho Chi Minh City, Vietnam

**Keywords:** ethics, intervention, pharmacist, pharmacy service, hospital, health-system, assessment tools

## Introduction

Pharmacist interventions (PIs), which are any actions by pharmacists that can directly change the patient management or therapies (1), play a crucial role in improving patient outcomes by mitigating adverse drug events. Multiple tools have been developed to assess the significance of these interventions, with the primary focus on the clinical and economic impacts (2). Typically, PI assessment is an independent process from PI conduct. In specific, after the pharmacists make the PIs, an expert (or group of experts) will review the PIs and assess them multidimensionally using pre-existing tools. These assessments may be used for revising the hospital policies or protocols to balance the hospital operations and patient outcomes. Despite progress in the development of PI-assessing frameworks, the ethical impact of PIs has largely been neglected in the current assessment tools. Ethical considerations are pivotal in healthcare decisions (3), yet remain underrepresented in evaluation frameworks for PIs. One contributing factor is that PIs are not medical orders but are considered consultations for healthcare providers. Hence, many believe PIs do not have direct ethical impacts on patient treatment or healthcare (4, 5).

However, ethical issues in healthcare require an inter-professional approach (6), including the active engagement of pharmacists. This poses an ethical requirement for PIs (7), especially regarding drug-related problems (DRPs). Given the limited inclusion of this aspect in the PI-assessing tools (2), there are concerns about the breadth of these frameworks. The lack of focus on incorporating the ethical dimension in existing tools hinders the recognition of pharmacist contributions, particularly in scenarios where ethical considerations are central to decision-making. In this paper, we aimed to highlight this issue and potential approaches to integrate ethics into assessment tools for PIs. While there are many approaches to exploring ethical considerations in clinical practice, this paper focuses on the core principles of medical ethics, i.e., autonomy, non-maleficence, beneficence, and justice (3). These principles are the minimum acceptable criteria to ensure ethical standards in clinical practice. Given the major role of health-system pharmacists in addressing DRPs (2), we only comment on DRP-targeted PIs. Pharmacist-initiated orders have stringent regulations and, thus, are outside the scope of most assessment tools. To avoid confusion, we also would like to re-emphasize that this paper does not imply any changes to the practice of health-system pharmacists but only focuses on revising the assessment tools for PIs.

## Clinical dimension

PIs are performed to address DRPs (2), therefore, the clinical impacts of PIs should correlate with the severity of DRPs (8), not a stand-alone index as in previously proposed assessment tools (2). For instance, in pain management for patients with cancer, overdoses (with a 20% increase in standard dosing) are flagged in a similar manner for both opioids and acetaminophen (also known as paracetamol). However, patient outcomes could differ significantly if these DRPs are not intervened. Addressing acetaminophen overdose is simply keeping the total dose per day below a well-defined threshold, e.g., 4 g for patients without liver impairment. However, it is more complicated to monitor and prevent overdose of medications without ceiling effect like opioids (9). Consequently, if we only use the current assessment tools to evaluate these overdosing DRPs, the 2 PIs should implicate similar clinical impact (resolving overdose). Compared with the PI for acetaminophen overdosing, the PI for opioid DRP is underestimated and implicitly documented as inadequate compliance with the beneficence principle of medical ethics.

More importantly, if the patients and providers are not in agreement on the goals of treatment, all medical orders and PIs should respect the autonomy of the patients (10). Disregarding patient choices or decisions can breed controversy over medical ethics (11). In such cases, PIs should be tailored partially to reflect the autonomy of the patients, given that they have sufficient decision-making capacity (11). Available PI-assessing tools cannot take this into account, as they only focus on the professional opinions of pharmacists but not the patients' perspectives (2). Typical examples are patient refusal of treatment in terminal illness or deprescribing in patients with limited life expectancy. Profession-driven PIs would focus on optimizing the active treatment, whereas patient-preferred PIs may only suggest palliative care due to the unnecessary burden of treatment. These types of dilemmas are normally outside the capacity of a PI-assessing tool. Therefore, assessing the clinical dimension of PIs without considering their clinical contexts and adjusting for patients' preferences can lead to ethical concerns and undermine the applicability of these tools.

## Economic dimension

While the economic dimension is primarily based on the hospital perspective, it should not bypass the non-maleficence and beneficence principles of medical ethics (12). In specific, cost savings of PIs are only meaningful if the initial medical orders or practices are professionally and ethically acceptable. For example, in a patient currently taking digoxin who develops hypokalemia (a common trigger of digoxin toxicity (13)), not correcting potassium level is often unacceptable. A PI attempting to supplement potassium for this patient, given no contraindication, is likely to be considered uneconomical by the hospital or payer perspectives. Such assessments should not be conducted based on clinical and ethical standpoints. This also applies to the PI-consulted medical orders or practices, meaning that if the PIs cannot ensure professionalism and ethics, the economic dimension should not be assessed as well.

## Organizational dimension

The lack of ethical considerations in the organizational dimension echoes that in the economic dimension. For instance, it is irrational to assign a negative organizational impact to a PI that separates the injections/syringes due to physicochemical incompatibility. In this case, the PI should not be discouraged in any manner despite an increase in workload and time for medication administration. Additionally, drug shortage-related issues create critical dilemmas for pharmacists. These are complex situations that require ethical and clinical justifications on a case-by-case basis (14). The foundational theory of managing drug shortages is balancing the quality of care for an individual patient and for a group of patients or society (15). Given this allocation trade-off, no PI-assessing tools can address the justice principle in these cases. Consequently, assessing the organizational dimension of the PIs can be ethically challenging in non-trivial scenarios.

## Ethics management and integration

The simplest solution to address ethical issues that arise during PI assessment is limiting the utilization of these tools for scenarios that can be exempted from significant ethical considerations. Of note, this approach cannot completely solve the problem, as the complex scenarios are still not properly covered. We suggest adding a dimension representing the DRP severity and integrating medical ethics into these tools, either inside other dimensions or as a separate ethical dimension.

Regarding the former option, criteria to assess ethical aspects should be given within each dimension of the original tools. If the initial medical orders/practices and PIs ensure the 4 minimal ethics principles (autonomy, non-maleficence, beneficence, and justice) (3), it is rational to assess all dimensions of the PIs. Otherwise, the dimension(s) requiring ethical considerations should not be assessed but instead, noted as not applicable. For the latter option, ethical considerations should be assessed after the DRP severity and clinical dimensions. If this ethical dimension does not flag any change, all other dimensions can be assessed accordingly. In contrast, when there is a positive or negative change, all other dimensions should not be considered for assessment.

To clarify the ethical dimension when assessing PIs, let us take a look at a scenario of an unconscious patient with terminal illness (microsatellite instability-high metastatic colorectal cancer) and no insurance/family members. The standard medical order initiates pembrolizumab. Given the poor prognosis for this patient, a PI suggests stopping the anticancer regimen. Appraising the PI as a cost-saving action can be unethical, if the PI is meant to avoid the costs on the hospital. In another hypothetically similar situation, the medical order attempts to avoid the standard anticancer regimen to reduce the treatment costs and workload, whereas the PI suggests initiating pembrolizumab. Considering the PI uneconomical can also be unethical, as the intention of the medical order, in this case, is not clinically and ethically justified. Therefore, to proceed with the assessment of economic and organizational dimensions of the PI without triggering these ethical issues, both the original medical order and PI need to be ethically appropriate. This shows why and how ethical dimension should be added to the PI assessment tools.
